# The Physicochemical Properties of Decellularized Extracellular Matrix-Coated 3D Printed Poly(ε-caprolactone) Nerve Conduits for Promoting Schwann Cells Proliferation and Differentiation

**DOI:** 10.3390/ma11091665

**Published:** 2018-09-09

**Authors:** Chung-Chia Chen, Joyce Yu, Hooi-Yee Ng, Alvin Kai-Xing Lee, Chien-Chang Chen, Yueh-Sheng Chen, Ming-You Shie

**Affiliations:** 1 Graduate Institute of Basic Medical Sciences, China Medical University, Taichung 40447, Taiwan; DAL06@tpech.gov.tw; 2 Linsen Chinese Medicine and Kunming Branch, Taipei City Hospital, Taipei 10341, Taiwan; 3 School of Medicine, China Medical University, Taichung 40447, Taiwan; u102001504@cmu.edu.tw (J.Y.); hooiyeen@gmail.com (H.-Y.N.); Leekaixingalvin@gmail.com (A.K.-X.L.); 4 3D Printing Medical Research Center, China Medical University Hospital, Taichung 40447, Taiwan; m0975371198@gmail.com; 5 Master Program for Biomedical Engineering, China Medical University, Taichung 40447, Taiwan; 6 Biomaterials Translational Research Center, China Medical University Hospital, Taichung 40447, Taiwan; yuehsc@mail.cmu.edu.tw; 7 Lab of Biomaterials, School of Chinese Medicine, China Medical University, Taichung 40447, Taiwan; 8 Department of Bioinformatics and Medical Engineering, Asia University, Taichung 40447, Taiwan; 9 School of Dentistry, China Medical University, Taichung 40447, Taiwan

**Keywords:** autologous nerve grafting, decellularized extracellular matrix, polydopamine, poly(ε-caprolactone) conduits, neuronal differentiation

## Abstract

Although autologous nerve grafting remains the gold standard treatment for peripheral nerve injuries, alternative methods such as development of nerve guidance conduits have since emerged and evolved to counter the many disadvantages of nerve grafting. However, the efficacy and viability of current nerve conduits remain unclear in clinical trials. Here, we focused on a novel decellularized extracellular matrix (dECM) and polydopamine (PDA)-coated 3D-printed poly(ε-caprolactone) (PCL)-based conduits, whereby the PDA surface modification acts as an attachment platform for further dECM attachment. We demonstrated that dECM/PDA-coated PCL conduits possessed higher mechanical properties when compared to human or animal nerves. Such modifications were proved to affect cell behaviors. Cellular behaviors and neuronal differentiation of Schwann cells were assessed to determine for the efficacies of the conduits. There were some cell-specific neuronal markers, such as Nestin, neuron-specific class III beta-tubulin (TUJ-1), and microtubule-associated protein 2 (MAP2) analyzed by enzyme-linked immunosorbent assay, and Nestin expressions were found to be 0.65-fold up-regulated, while TUJ1 expressions were 2.3-fold up-regulated and MAP2 expressions were 2.5-fold up-regulated when compared to Ctl. The methodology of PDA coating employed in this study can be used as a simple model to immobilize dECM onto PCL conduits, and the results showed that dECM/PDA-coated PCL conduits can as a practical and clinically viable tool for promoting regenerative outcomes in larger peripheral nerve defects.

## 1. Introduction

The nervous system is a vast network which regulates and influences our bodily functions, including autonomic, sensory and motor functions [1]. Hence, if there are severe injuries to any part of the nervous system, there will be drastic changes to a patient’s quality of life. Our nervous system can be categorized into two parts, one is the central nervous system (CNS) and the other is the peripheral nervous system (PNS) [2]. The CNS is composed of the brain and the spinal cord, while the PNS involves any motor and sensory systems that are outside the brain and spinal cord. Statistics have shown that 2.8% of patients remain affected by peripheral nerve dysfunction post-treatment, and most of them end up having functional disorders, neurological pain, poor quality of life or even irreversible disabilities. Peripheral nerve damage may be caused by external trauma, or even during surgical procedures. In addition, the extent of the damage or injury to the nerve determines the difficulty of treatment. The distal end of the neuron would gradually degenerate after any traumatic damage to the nerves, while the proximal end would grow up to 1~2 mm/day for regeneration [3]. Neurons often fail to regenerate if the nerve damage extends beyond 15 mm. Therefore, until today, nerve rehabilitation and repair have still remained a continuous challenge in the field of regenerative medicine. Peripheral neuron regeneration after nerve trauma has always been an important topic of concern. The process of neuron recovery is a very complex and slow process. Current methods of treating nerve injuries include autografts and xenografts; however, these methods still involve issues of immunological rejection, which impede neuron regeneration [4]. Furthermore, there are very limited sources available for autologous grafting, and such grafting often includes additional grafting surgical procedures, and these procedures often involve the risk of exacerbated tissue damage and loss of function to the grafted area. Thus, utilizing methods of tissue bioengineering to replace grafting as a method of treating nerve injuries has become an emerging and significant focus of research within the field of nerve rehabilitation [5]. In this context, various research has emerged that is attempting to solve the problems of nerve rehabilitation by using nerve conduits [6]. 

The main function of nerve conduits is to provide structural support as a guiding passage for neuron regeneration. Hence, ideal nerve conduits should possess good biocompatibility and flexibility; they should also support adequate diffusion of oxygen, metabolites, and cytokines between peripheral tissue and the inner part of the conduits [7]. Autologous conduits, biodegradable conduits (such as those that are made out of Gelatin, PLGA, PCL, Chitosan), and polymer conduits (such as those that made out of stainless steel and silicon) are commonly used in clinical settings [8,9]. Both PLA and PCL are FDA-approved biomaterials, with PCL and PLA both having been individually extensively reviewed for biomedical applications. In this study, we chose PCL as the base material for our nerve conduit. However, some studies have proved that some acid product is released from PLA materials during degradation processing. On the other hand, PLA has a higher melting point compared with PCL, which presented a fragile behavior with high elastic modulus and low elongation at break. With this in mind, it was suggested that PLA is a more suitable candidate for bone engineering, while PCL would be a better candidate for soft tissue [10]. Based on the above reasons, our team chose PCL as our base material for this study. Although numerous research and animal studies have been conducted in the past to show the adequacy of the above-mentioned conduits in the replacement of autologous grafting, in reality, the results from clinical trials have not been as promising and effective as such research suggested [8,9]. A possible reason to explain this phenomenon could be attributed to the post-implantation unknown effects induced by foreign bodies, hence hampering the process of nerve regeneration. Nevertheless, numerous clinical trials of various conduits are still being conducted today [11]. In recent years, a novel approach of using decellularized extracellular matrix (dECM) as a material has emerged, and a great deal of research has been attempted, implementing them into the development of biomaterials for nerve conduits and as a material for tissue regeneration [12,13]. dECM is commonly obtained by processing healthy tissues/organs with chemical or physical methods to remove redundant cellular components, thus only retaining structural proteins, glycosaminoglycans and various growth factors that mimic the microenvironment of native tissues [14,15]. The dECM is thus able to provide a natural and similar microenvironment for cells to thrive in. With this concept in mind, current research has focused on developing a dECM-coated biomaterial that has both structural and biological behaviors which are very similar to the native environment [16,17].

Polydopamine (PDA) is one of the ideal choices as an adherence promoter to prevent segregation of dECM and substrates, and it can also be used to improve the interface binding strength of dECM and substrates [18]. Dopamine (DA) is a small molecule which is able to mimic the adhesive protein of marine mussels. It has the chemical structure of catecholamines when exposed to a certain pH (through oxidative transformation from catechol to quinone) [19]. Therefore, this material-independent PDA-coating process was very easily, quickly, and efficiently achieved through base-triggered oxidation and polymerization of DA for surface functionalization of various substrates via a single-step process. This PDA ad-layer can be used as a platform for surface modification, including spontaneous deposition of different factors, as well as covalent immobilization of various growth factors and proteins [20,21]. A recent novel strategy to improve the physiological properties of various biomaterials involves the admixing of biological molecular dopamine (DA) and polydopamine (PDA) into the formulation [22]. The modifications of the hydrophilic surface and the functional groups were shown to promote cellular behaviors on self-assembled PDA nano-layers on the bioceramic biocomposites [23]. Kao et al. fabricated and functionalized a 3D-printed PLA porous scaffold and fabricated the mussel-inspired coating process to enhance stem cell behaviors [24].

The aim of this study was to fabricate homogenous dECM and PDA-coated 3D-printed PCL-based conduits. PCL is an FDA-approved synthetic biomaterial that is commonly used for neural tissue engineering conduits or scaffolds, and has been reported to have sufficient mechanical properties and stable biodegradation. However, the bioactivity and inertness of PCL could still be further enhanced using additional methods such as surface modifications or hybridization of PCL with other biomaterials. The initial PDA-coated PCL conduits function as a modified supportive material, which would be subsequently coated with dECM from the nerve. The mechanical properties and chemical composition were analyzed using the EZ test and electron spectroscopy for chemical analysis (ESCA). The dECM-coated PDA/PCL conduits influence adhesion, proliferation and differentiation of primary rat Schwann cells.

## 2. Materials and Methods

### 2.1. PCL Conduits Fabrication

**Figure 1:**
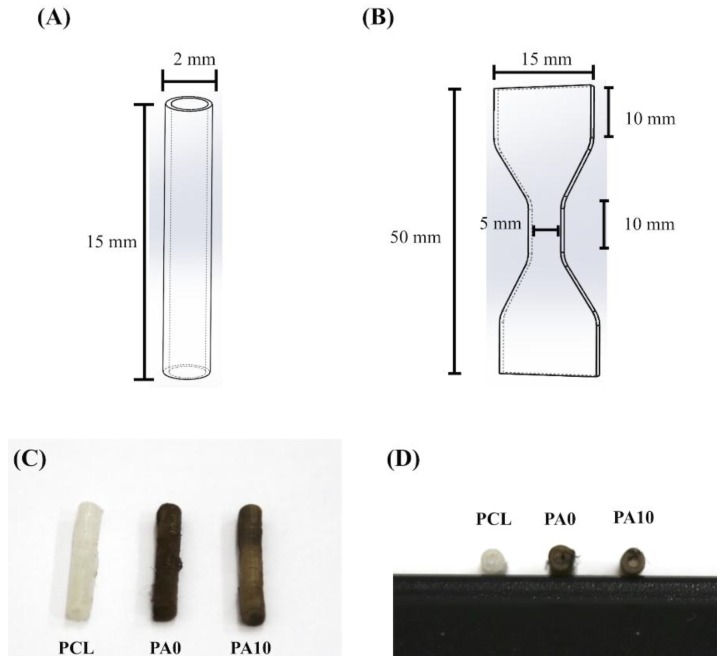
Schematic drawing of (**A**) the conduit and (**B**) the dumbbell-shaped sample used in the mechanical testing. The (**C**) top- and (**D**) side-view photograph of dECM/PDA-coated conduits.

For this study, we used commercially available PCL filament (eMate PCL, eSUN, Taipei, Taiwan) with a diameter of 1.75 ± 0.10 mm. The nerve conduit was printed by commercial self-assembly 3D printer (Prusa i3 MK8, Sintron, Taipei, Taiwan). Solid Work software was used to design our scaffold with the following parameters: 15 mm length, 2.0 mm inner diameter and 2.5 mm outer diameter (Figure 1A). Following which, we used the slicing software Slic3r to convert the predesigned stl-file to a g-code file. The following parameters was used to fabricate our 3D-printed PCL conduits: 400 µm diameter nozzle, printing speed of 10 mm/s, 80 °C for melting of PCL filaments. PCL fibers were then deposited layer-by-layer until our desired conduits were formed. The thickness of each layer was pre-determined to be at 100 µm.

### 2.2. Mechanical Properties of 3D-Printed PCL Conduits

The tests for mechanical properties were done in dry environments. The maximum tensile force was analyzed using a universal tensile tester. For this test, we had the samples printed out to be in a dumb-bell shape and the samples were subsequently stretched from both ends at a rate of 1 mm/min (Figure 1B). Six assays were conducted for each sample and the average was recorded.

### 2.3. Nerve Decellularization

Rat sciatic nerves were dissected and removed as a whole section with approximately 4 mm in length and soaked with distilled water for 15 min. The nerves were then treated with 0.5% Triton X-100 in 1 M NaCl distilled water solution for 2 h with constant stirring. Subsequently, the nerves were treated with 1% SDS in 1 M NaCl distilled water solution for two days with constant stirring. The solution was changed once daily for this step. After which, the nerves were sterilized by 4 h treatment of 0.1% peracetic acid and 4% EtOH in 1 M NaCl distilled water solution. After which, the decellularized tissues were washed with distilled water for 4 h to wash off any remaining detergent and solutions. Decellularized tissues were lyophilized for 24 h and kept in −20 °C Celsius till further usage.

### 2.4. Nerve dECM/PDA-Coated Conduits

The direct soaking method was used to deposit the PDA onto the PCL conduits. Simply, all conduits were washed with sterilized PBS, followed by a 12 h immersion in dopamine solution (1 mg/mL in 10 mM Tris, pH 8.5) while being placed on a 30 rpm shaker at room temperature. After which, the conduits were removed from the solution and rinsed several times with PBS. At the same time, 0.5 M acetic acid and pepsin was used to dissolve and digest the frozen dECM powder. The pepsin used in this totaled 10% of the total dECM weight, and the solution was stirred at room temperature for 4 days. Following which, the dECM solution was centrifuged at 5000 rpm for a duration of 15 min. This was to remove any undissolved dECM before adjustment of pH to 7.4 with 5 M NaOH. Then we put the PDA-coated PCL conduits into the dECM solution with different concentrations (0, 5, and 10 mg/mL) under shaking at 25 rpm at 4 °C for 12 h. An electron spectroscope was used to analyze for chemical compositions and elemental compositions of our dECM/PDA-coated PCL specimens (ESCA, PHI 5000 VersaProbe, ULVAC-PHI, Kanagawa, Japan). The concentrations of the analyzed compositions are presented in atomic percentage. In addition, water contact angle test of each conduit was done under room temperature. Briefly, the samples were placed on top of a platform and 5 μL of MilliQ water (Millipore, Danvers, MA, USA) was then pipetted onto the surface of each sample. A camera was used to capture images of the water droplet after 20 s. After which, ImageJ 1.45 software from the National Institutes of Health (Bethesda, MD, USA) was used to analyze and measure the water contact angle.

### 2.5. Biocompatibility of dECM/PDA-Coated Conduit Extracts

Indirect biocompatibility was analyzed using a revised version of ISO10993-12. Briefly, we printed 6 cm^2^ dECM/PDA-coated conduits as our samples, which were subsequently washed thrice with PBS, followed by sterilization in 75% ethanol at room temperature in a laminar flow for 30 min. To obtain the extracts of our dECM/PDA-coated conduits, the conduits were then immersed in Dulbecco’s modified Eagle’s Medium (DMEM, Invitrogen, Waltham, MA, USA) and placed in a 37 °C incubator with settings of 75% humidity and 5% CO_2_ for 24 h. Concurrently, cell culture of L929 cells with a cell count of 104 cells were seeded in a 96-well plate at 37 °C for 24 h. Following which, DMEM was removed and replaced with 200 µL/well of dECM/PDA-coated conduits extract solution. After 1 day of cell culture, the extract solution was removed and replaced with 100 µL of MTT solution (5 mg/mL) in each well. Following which, the culture was then left to be incubated for 3 h in the dark. Then, MTT solution was removed and replaced with 100 µL of dimethyl sulphoxide for dissolution of formed formazan by MTT solution. A microplate reader (Infinite 200^®^ PRO microplate reader, Tecan, Männedorf, Switzerland) was used to analyze for absorbances of each well.

### 2.6. ELISA Analysis

To analyze the collagen I (Col I) and laminin concentration in the dECM/PDA-coated conduits extract, we used an ELISA for analysis. An enzyme-linked immunosorbent assay kit (Invitrogen) was used to consider the levels of Col I and laminin by following the instructions from the manufacturer. Subsequently, the levels of Col I and laminin were determined by correlation with a standard curve. Six independent experimental analyses were performed for each specimen.

### 2.7. Cell Adhesion

All our dECM/PDA-coated PCL specimens were sterilized in 75% ethanol concurrently under exposure of ultraviolet light for 30 min. The primary rat Schwann cells (RSCs) used in this study were obtained from ScienCell Research Laboratories (San Diego, CA, USA) and cultured in Schwann cells medium (#1701, Sciencell) to cell passage 3–8. A density of 105 cells per specimen of RSCs were directly seeded onto the specimens and placed in a 37 °C, 5% CO_2_ atmosphere incubator for various periods of culture time. After 3 and 6 h of culture, PrestoBlue^®^ (Invitrogen, Waltham, MA, USA) reagent was used to evaluate and analyze for cell quantities. Briefly, the culture medium was removed and the specimens were rinsed several times with cold PBS. Following which, a ratio of 1:9 PrestoBlue^®^ and fresh medium were used to fill each specimen. Subsequently, they were incubated for 90 min at 37 °C before measurement of PrestoBlue^®^ absorbance. In brief, the reaction solutions were removed from the specimen and placed in a new 96-well plate. Absorbance was measured by using a Tecan Infinite 200^®^ PRO microplate reader at 570 nm with a reference wavelength of 600 nm. Data for this study were obtained in triplicate from three different cultures, and RSCs cultured on culture plates without any specimen were used as a control (Ctl). 

For protein assays, the seeded RSCs were lysed with NP40 buffer (ThermoFisher, Waltham, MA, USA) after 3 h of cell seeding. BCA protein assay kit was used to analyze for total protein concentrations. Briefly, cell lysates (40 μg protein/sample) were segregated using sodium dodecyl sulfate-polyacrylamide-polyacrylamide gel electrophoresis before being transferred over to PVDF membranes (Millipore, Danvers, MA, USA). 2% bovine serum albumin (Invitrogen, Waltham, MA, USA) in tris-buffered saline with 0.1% Tween 20 were used to fixate the proteins on the membranes before immunoblotting with primary anti-focal adhesion kinase (FAK), anti-phospho-FAK (p-FAK), and β-actin (GeneTex, San Antonio, TX, USA) antibodies for 2 h. Following the immunoblotting, the membranes were incubated with horseradish peroxidase (HRP)-conjugated secondary antibodies to allow chemiluminescence and physical visualization of bands with an ECL detection kit (Invitrogen). For capturing data from chemiluminescent Western Blots system by cooled CCD cameras (Fusion Solo Vilber Lourmet system, Paris, France). Then, we confirmed the signal strength and detected the target band signal for different exposure time (3–5 min). The optical densities of the bands of digital images were quantified using Gel-Pro Analyzer software 3.0 (Media Cybernetics, Silver Springs, MD, USA). For each protein, expression levels were normalized to β-actin. 

Also, F-actin staining was conducted for cell morphology observations. Briefly, the specimens were rinsed thrice with cold PBS before being fixate with 4% paraformaldehyde for 20 min. Following which, the cells were placed with 0.1% Triton X-100 at room temperature for 15 min to permeate the cells. Then, the RSCs were incubated with AlexaFluor-594-conjugated phalloidin for 1 h and DAPI (4′,6-diamidino-2-phenylindole, dilactate) was used to stain for nucleus. For DAPI, the fixated cells were placed in DAPI for 20 min in the dark at room temperature. The images were photographed using the Leica TCS SP8 X white light laser confocal microscope (Leica Microsystems GmbH, Wetzler, Germany). Finally, the total areas of the red label cell of the images were analyzed using ImageJ (Bethesda, MD, USA).

### 2.8. Statistical Analyses

For all data and statistical analysis for this study, all data were retrieved and gathered by the same observer and subsequently expressed as the mean ± standard deviation (SD). SAS 9.4 was used to conduct inter-group comparison using t-test of variance with a statistical significance of *p* < 0.05. (SAS Institute, Inc., Cary, NC, USA).

## 3. Results and Discussion

### 3.1. The Characterization of PCL Conduits

**Figure 2:**
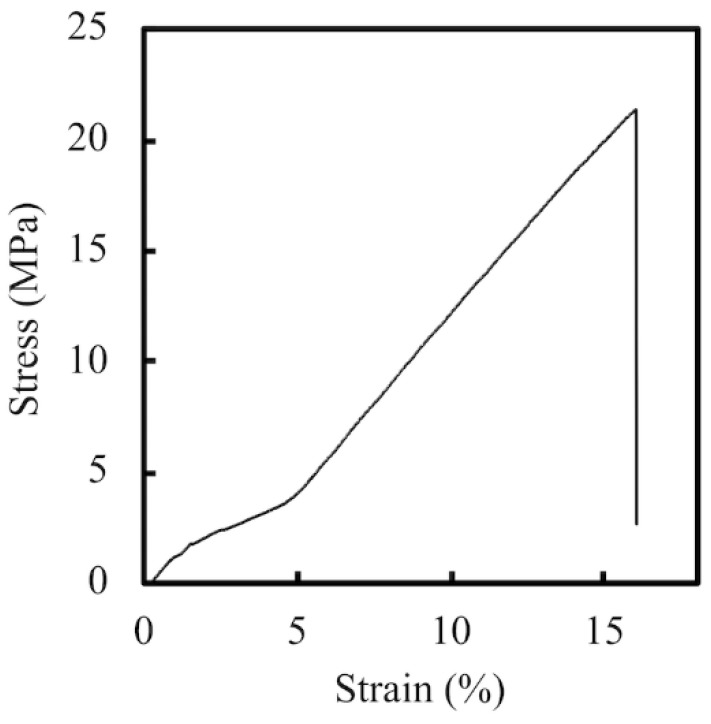
The tensile stress–strain curve of 3D-printed PCL specimens.

The PCL- and PDA-coated conduits are white and dark brown in color, respectively (Figure 1C,D). Representative stress–strain curves of PCL conduits at the same strain rate of 0.02/s are shown in Figure 2. The PCL conduit was transparent and concentric with both smooth inner and outer lumen surfaces. The maximum tensile strength and the elastic modulus of the 3D-printed PCL conduits was 21.2 ± 3.2 MPa and 142.5 ± 9.4 MPa. During the tests, for all specimens, the conduits were all torn and fractured at the middle of the conduits without any differences being found in locations between the samples. In previous studies, it was demonstrated that the elastic modulus of the human nerve was similar to the stress–strain behavior of rat nerves (13.8 ± 5.4 MPa) [25,26]. In our study, it was found that PCL conduits had significantly higher mechanical properties as compared to sciatic nerves. This is an advantageous property to have, as it allows more room for handling and suturing during surgical processes. Our data clearly indicated that the PCL conduits were able to maintain such mechanical properties that would allow this kind of manipulation during surgeries. 

**Figure 3:**
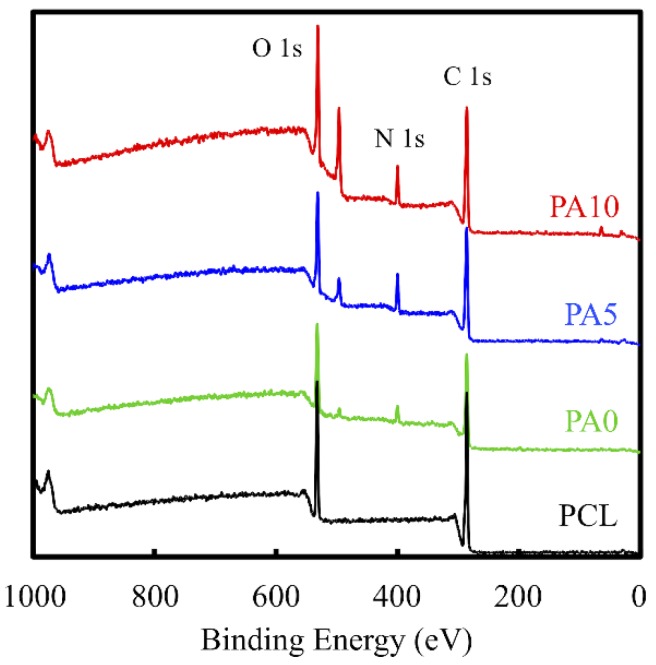
XPS spectra obtained on PCL conduits after coating with PDA and various concentrations of dECM. The peak at 400 cm^−1^ corresponds to the N1s from the amine group of PDA. The increase of the N–C=O component at 289.0 eV indicates the introduction of maleimide and ECM.

The deposition of dECM/PDA on PCL is also supported by the XPS high-resolution spectra (Figure 3). The photoelectron peaks of the PA0, PA5, and PA10 appear, along with the emergence of N1s at 400 eV. After PDA deposition, the amount of nitrogen was much greater than those seen with the pure PCL that indicated PDA coated on the PCL surface. In addition, the increase of the N–C=O component at 289.0 eV indicated the introduction of maleimide and ECM bonds on the surface [27]. 

**Figure 4:**
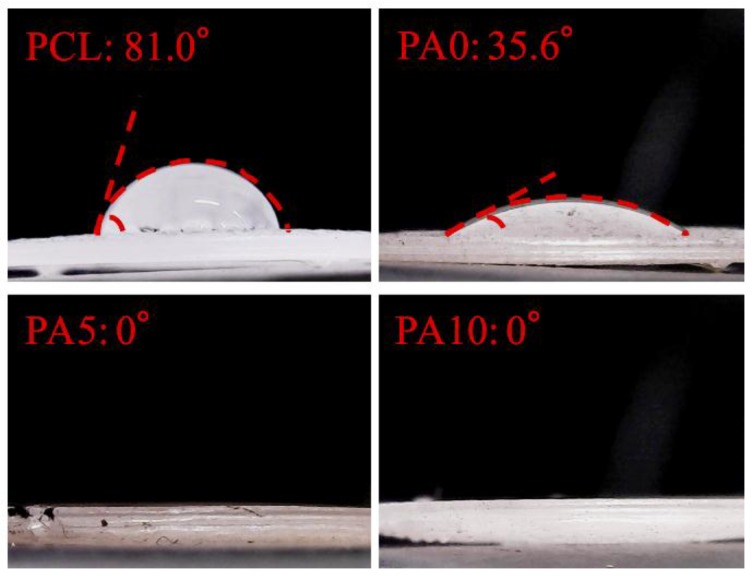
Water contact angle of various concentration dECM/PDA-coated PCL specimens.

Figure 4 shows the contact angle formed between the ddH_2_O droplet and the surface of the dECM/PDA-coated PCL substrate. The angle formed on the PCL material (81.0°) is higher than that of the PDA-coated PCL (35.6°). We deduced that the hydrophobicity of the PCL materials was affected by the presence of PDA [19]. Our result is consistent with previous studies and various literature confirms that PDA nano-layers are more hydrophilic than PCL substrates [28]. In addition, the dECM-coated group displayed the lowest contact angle (0°), which clearly indicated that the hydrophobicity of the dECM/PDA-coated PCL was further significantly promoted after dECM coating. Cai et al. demonstrated that the quicker spreading of water could be due to the relatively larger amount of embedded hydrophilic dECM components [29]. Cellular adhesion ability can be positively influenced if the cells are seeded on a scaffold with a water contact angle lower than 80°. Overall, these result show that the PCL-based scaffolds were more hydrophobic and that substrates coated with dECM were extremely hydrophilic [30].

### 3.2. Cytotoxicity of dECM/PDA-Coated PCL Conduits

**Figure 5:**
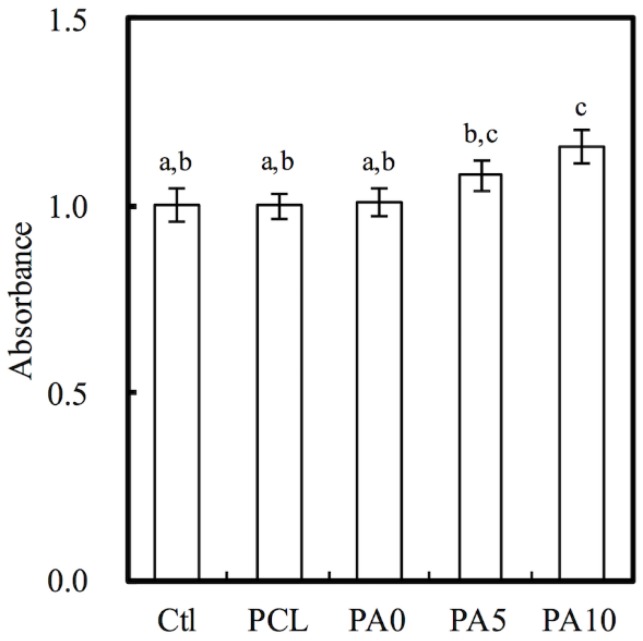
Quantification of cytotoxic test of extract solutions of dECM/PDA-coated PCL conduits relative to controls on rat Schwann cells. Values not sharing a common letter are significantly different at *p* < 0.05.

Indirect cytotoxicity testing using MTT assays is an important method for evaluating the cytotoxicity of conduits, especially so for evaluating the cytotoxicity of components that were gradually released by the conduits that followed ISO 10993-12. The results of the MTT test were presented as the percentage of viable L929 cells in the presence of different concentrations of dECM/PDA-coated PCL conduits and neat PCL scaffolds as compared to the controls, which represent a pure cell culture of L929 cells (Figure 5). The cytotoxic assay showed that all 3D-printed PCL conduits were non-toxic to L929. There was no significant difference of the cell viability between Ctl, PCL and PA0, which indicates the PCL-based nerve conduits were suitable for cell culture. Interestingly, the cell number of PA10 was significantly higher (*p* < 0.05) than Ctl, PCL and PA0, with an approximately 15% increment. It is worthy of note that there were increased cell metabolites detected by MTT assay in all groups as compared to Ctl, and cell proliferation was enhanced according to the dose-dependent increment of dECM concentrations, especially with 10 mg/mL dECM. This caused positive alterations in cell behaviors, rather than apoptosis or functional impairment, and thus molecules released from dECM, such as Col I and laminin, might have a role to play in enhancing cell proliferation [31]. 

### 3.3. The Nerve-Related Growth Factor of dECM Coating

**Figure 6:**
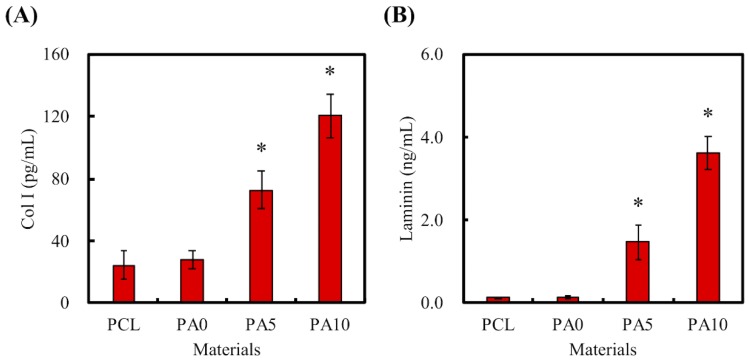
(**A**) Col I and (**B**) laminin were secreted by dECM/PDA-coated PCL conduits after immersion in DMEM for 3 h. * indicates a significant difference (*p* < 0.05) compared to PA0.

As mentioned, ECM is a naturally occurring protein network that provides precise structural support and guidance for cellular activities. The presence of laminin and Col I coated onto our PCL conduits was shown and quantified using ELISA protein assays and is presented in Figure 6. As seen in Figure 6A, the amount of Col I was significantly higher on PA5 and PA10 as compared to PA0 and PCL (*p* < 0.05). The laminin amounts on PCL, PA0, PA5, and PA10 were 0.13 ± 0.02 ng/mL, 0.13 ± 0.03 ng/mL, 1.47 ± 0.41 ng/mL and 3.62 ± 0.42 ng/mL, respectively (Figure 6B). The overall results showed that dECM could be effectively coated onto the PCL conduits through the assistance of PDA as an adherence promoter, and that the amount of dECM coated could be increased exponentially with the dose-dependent increment of the dECM concentrations. In addition, it assists in relaying biological signals to cells, as ECM has specific binding sites for various substrates and can also act as a reservoir for soluble factors bounded to ECM components [32]. Of these, laminins are the most significant class of ECM proteins in the nervous system, and they have been reported to have critical roles in supporting various functions, such as neuronal migration, axonal outgrowth, myelination and formation of neuromuscular junctions [33]. On the other hand, collagens are the most abundant and critical ECM proteins, mainly functioning as structural anchoring points for various enzymes or proteins.

### 3.4. Cell Adhesion, Cell-Adhered Related Protein and Cell Morphology

**Figure 7:**
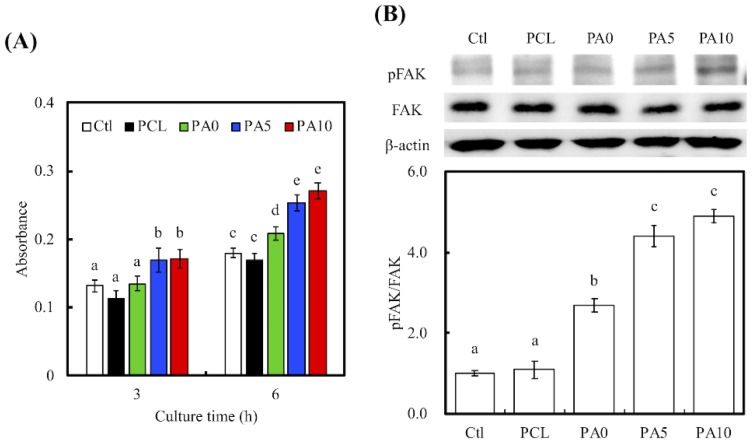
(**A**) The adhesion of RSCs cultured with various dECM/PDA-coated PCL conduits for 3 and 6 h; (**B**) The pFAK expression of cells cultured on specimens for 3 h. The values shown are means ± standard errors for all the assays. Values not sharing a common letter are significantly different at *p* < 0.05.

Cellular adhesion on the various conduits was measured using Prestoblue assays (Figure 7A). After 3 h of cell culture, the absorbance of both PA5 and PA10 was significantly higher than that of Ctl, PCL and PA0 (*p* < 0.05). However, there were no significant differences between PA5 and PA10, and there were also no significant differences between Ctl, PCL and PA0. The absorbances of the dECM/PDA-coated PCL conduits increased exponentially with the amount of dECM coatings, with PA10 having an increment of approximately 40% after 3 h of culture. However, there was a decrease in the absorbance of PCL as compared to Ctl and the rest of the groups. Similarly, there was a decrease in the absorbance of PCL after 6 h of culture, but there remained no significant differences between PCL and Ctl (*p* > 0.05). It was also worthy of note that after 6 h of culture, all dECM/PDA-coated PCL conduits had significantly higher absorbances than PCL and Ctl. Degree of cellular adhesion is commonly used as a determinant factor in predicting subsequent levels of cellular proliferation and differentiation. Combinations of natural proteins or polysaccharides with synthetic polymers have commonly been reported to enhance cell adhesion and proliferation in comparison to neat polymers [34]. 

To further quantify the level of cell adhesion, the levels of phosphorylated focal adhesion kinase (pFAK), focal adhesion kinase (FAK) and β-actin expressions were also examined using western blot (Figure 7B). RSCs cultured on PA5 and PA10 had a higher expression of pFAK than those in the other groups, with no notable differences in expression of FAK and β-actin. In addition, quantitative data of pFAK/FAK ratio showed that there were increases by approximately 2.45-, 4.01- and 4.46-times for PA0, PA5 and PA10, respectively, as compared to PCL. Similar to the above studies, significant differences were present between PA5, PA10 when compared to the rest of the groups. In this quantitative study, PA0 was found to have notable significant differences when compared with Ctl and PCL. These results clearly showed an indication of the determining role of dECM on focal adhesion contacts. Cell signaling cascades were generally triggered in the presence of cell–ECM interactions, of which there would an up-regulation of growth factors as an end result. FAK is activated by binding directly to the cytoplasmic domain of integrin β1 at focal adhesion sites. Studies have identified FAK as an important mediator of integrin signaling, and such integrin-mediated cell adherence promotes auto-phosphorylation of FAK [35]. After which, pFAK regulates focal-contact formation, cell spreading and induction of signaling pathways that are essential for cell motility [36]. pFAK was also reported to further activate extracellular signal-regulated kinase (ERK), which enhances VEGF expression and cell proliferation [37]. Therefore, pFAK is often used as a significant biomarker for cell/material interactions due to their important role in cell adhesion complex remodeling. Similarly, the increased pFAK expression in the PDA/dECM PCL conduits was closely correlated with the increased cellular adhesion as shown in Figure 7A and further supplemented by the increased laminin and Col I coatings found on the conduits. Interestingly, these results were consistent with the observations made by others, whereby combination of natural proteins or polysaccharides with synthetic polymers were able to enhance cell adhesion [38]. In fact, RSC had a rapid response to axonal injury, with an estimated time of response prior to the beginning of axonal degeneration. They recruit macrophages by releasing chemokines and cytokines to aid in improving the rate of myelin clearance. Our data support that the 3D-printed PCL nerve conduits act as a bridge and shorten the gaps between two blunt nerve fiber endings [39].

**Figure 8:**
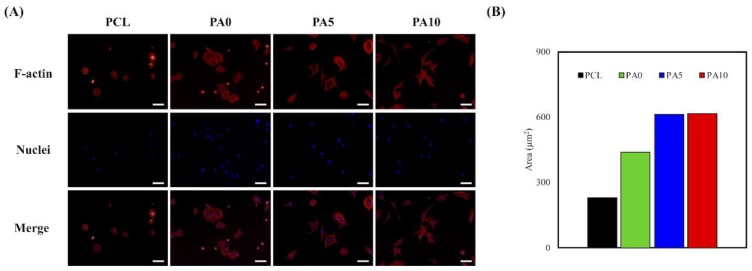
(**A**) F-actin (red) and nuclei (blue) staining; and (**B**) the area quantification the area of RSCs seeding on dECM/PDA-coated PCL substrates for 3 h. The scale bar is 200 µm.

To further supplement the above findings, F-actin stains and DAPI immunofluorescence images were used to visualize and observe cellular morphology after adhesion and spreading. As seen in Figure 8A, RSCs on the neat PCL conduits were in a small spherical shape, which was a clear indication of a typical non-adhered morphology. In comparison, cells on PA10 exhibited a flattened and elongated morphology, with F-actin stains clearly indicating cell membrane proteins and widened spreading. The areas of red cells on PCL, PA0, PA5, and PA10 are 230.25 µm^2^, 438.73 µm^2^, 611.46 µm^2^, and 613.91 µm^2^, respectively (Figure 8B). In addition, there was an increased number of cells when compared to neat PCL conduits. These data further supported the findings above, in which that surface modification with dECM contributed significantly to the fabrication of functional PCL conduits that were non-cytotoxic and were able to enhance cell adhesion, which would further influence cell proliferation and differentiation.

### 3.5. Cell Proliferation and ERK Expression

**Figure 9:**
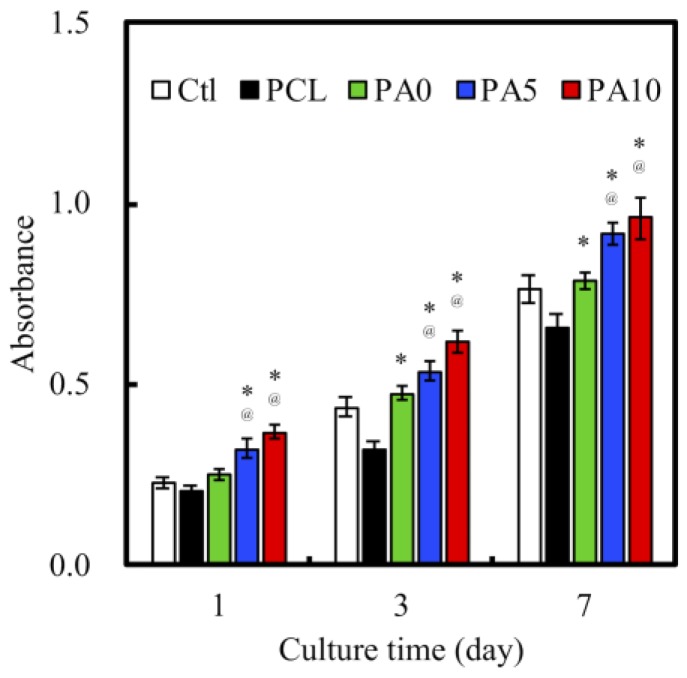
Proliferation of RSCs cultured on various dECM/PDA-coated PCL conduits for 1, 3, and 7 days. * indicates a significant difference (*p* < 0.05) compared to PCL. @ indicates a significant difference (*p* < 0.05) compared to PA0.

Quantification of cell proliferation over three various culture times of 1, 3 and 7 days was measured. As shown in Figure 9, there was a constant increase of absorption values over the 7 days for all groups. This showed that there was a constant and steady proliferation over this period, with a higher cell proliferation on the dECM/PDA-coated PCL conduits. There was a consistent significant difference between PA5 and PA10 when compared to Ctl and PCL over all time points (*p* < 0.05). There were no significant differences between PA5 and PA10 for all time points. Similar to the findings above, neat PCL conduits were found to have a lower proliferation rate compared to Ctl. In conjunction with the results of the proliferation assay and the results reported above, it can be postulated that the dECM coating plays an important role in modulating cell behavior resulting from the increased synergistic combination of structural proteins and PDA, and that higher concentrations of dECM coatings of 5 and 10 mg/mL were effective in supporting cell adhesion and proliferation through higher expression of growth factors.

**Figure 10:**
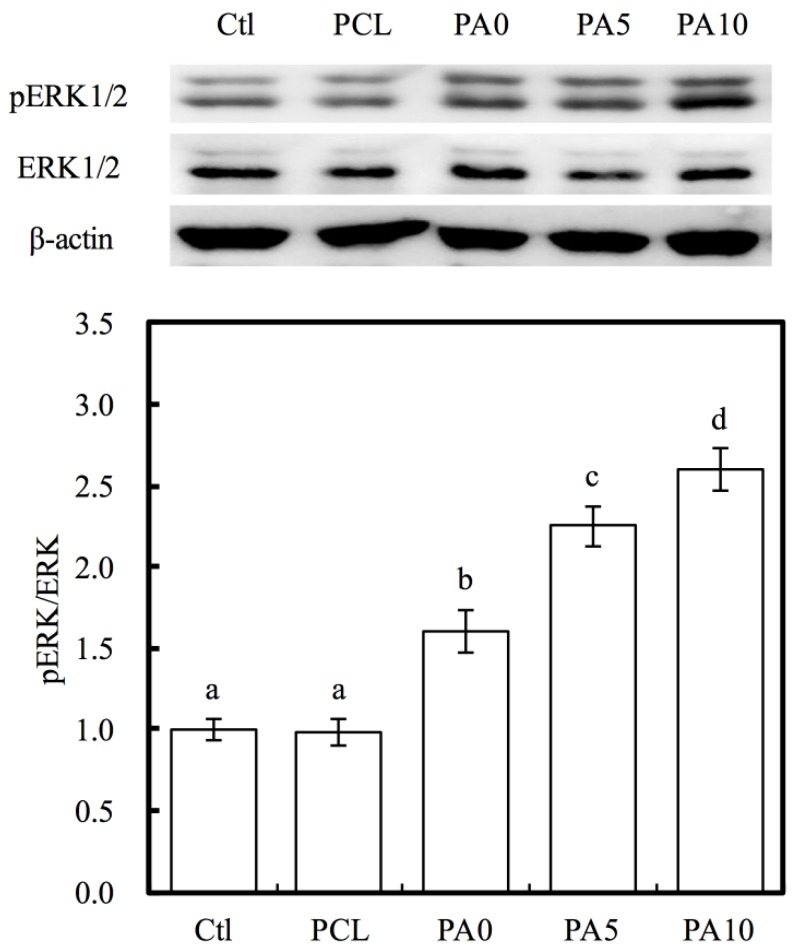
Western blot data showing various dECM/PDA-coated PCL conduits promote pERK levels in RSCs. The values shown are means ± standard errors for all the assays. Values not sharing a common letter are significantly different at *p* < 0.05.

As seen in Figure 10, ERK1/2 activation was observed by western blot analysis, and this was evidenced by the increase in pERK1/2 expression. In addition, quantitative analysis of pERK/ERK ratio indicated that there were significant increases of 63%, 130% and 166% of PA0, PA5 and PA10, respectively, when compared to PCL. The mitogen-activated kinase (MAPK) is a major signaling pathway for transmission of a broad range of growth factors and extracellular signals that influenced cellular activities such as proliferation, differentiation and influencing cell cycle [40]. In this pathway, ERK1/2 plays a huge role in being an activator of this pathway and they act as ligands for G protein-coupled receptors, cytokines and other mitogenic factors. Increased ERK1/2 expression was recently reported to regulate and increase myelin thickness in the nervous system, thus suggesting that ERK1/2 was an important factor in the maintenance of the integrity of myelin and axons in the nervous system [41]. Active ERK1/2 (pERK1/2) can positively or negatively regulate several downstream transcription factors by regulating a wide range of kinases to regulate cellular activities. Similarly, recent studies have reported that chronic up-regulation of pERK1/2 is strongly associated with corneal fibrosis [42]. Despite the lack of details regarding the precise mechanism behind the proliferative roles of pERK1/2, these results are consistent with the above findings that dECM/PDA-coated PCL conduits are able to enhance cell adhesion and proliferation after functional surface modifications. In addition, pERK1/2 have commonly been used as a significant marker due to their important role in cell proliferation, and more recently, as a marker for myelination and axonal regenerations [43].

### 3.6. Neural Differentiation

**Figure 11:**
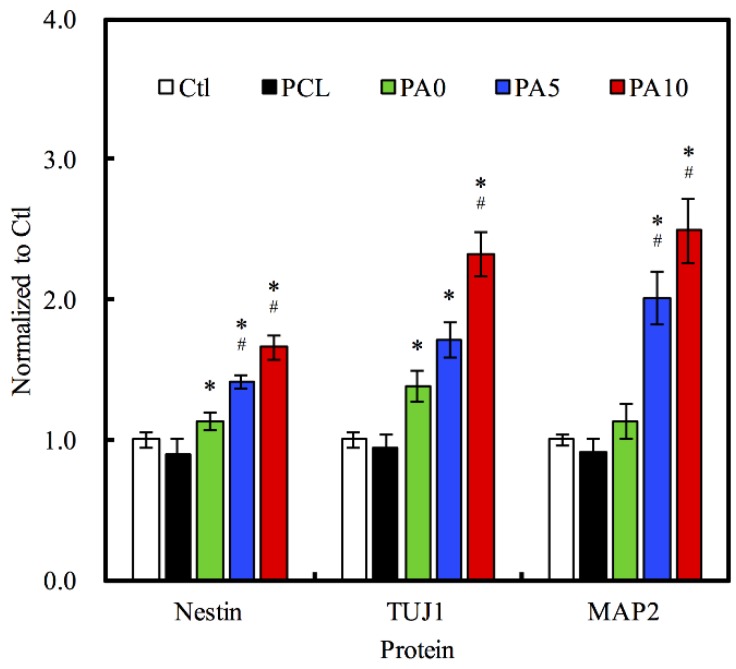
Effects of dECM/PDA-coated PCL conduits on the neural differentiation-related proteins expressions of Nestin, TUJ1, and MAP2. * indicates a significant difference (*p* < 0.05) compared to PCL. # indicates a significant difference (*p* < 0.05) compared to PA0.

Nestin, TUJ-1 and MAP2 were three neuron-specific markers that have commonly been used for establishing the efficiency of RSC differentiation. In our study, levels of Nestin, TUJ-1 and MAP2 were analyzed using ELISA as a determinant of the efficacy of the use of dECM/PDA-coated conduits (Figure 11). There were significant differences observed in the expression of Nestin, TUJ1, and MAP2 for both PA5 and PA10 as compared to the other groups. On the other hand, PA0 only had significant differences in the expression of Nestin and TUJ1 as compared to the rest. Specifically, on PA10 conduits, we can see that Nestin expressions were 0.65-fold up-regulated, while TUJ1 expressions were 2.3-fold up-regulated and MAP2 expressions were 2.5-fold up-regulated as compared to Ctl. These results demonstrated that higher concentrations of dECM coating were able to enhance differentiation of RSC into neural cells during the same culture time frame when compared to that of Ctl. Nestin and TUJ1 are recognized as early- to mid-phase protein markers, while MAP2 is known to be a late-phase protein marker. TUJ1 is part of the microtubule family and has a huge role to play in maintaining intracellular axonal transport and maintenance of intracellular structures. On the other hand, Nestin is a type of intermediate filament that is mostly unique to RSCs and therefore can be considered as a RSC specific marker [44]. Specifically, TUJ1 can only be found to be different in the early phases of neuronal cell differentiation, and thus is known as an early-phase protein marker. Together with Nestin, both TUJ1 and Nestin are commonly classified as neuronal differentiation markers, whereby their levels of expression would be greatly down-regulated after neuronal differentiation and maturation [45]. On the other hand, MAP2 is a protein found only in the cytoskeleton of mature neural cell, and thus RSCs usually have a low expression of MAP2 during the early stages of differentiation. A gradual increment of MAP2 can be seen as neuronal cells mature and progress throughout the neuronal development process. Therefore, our results were consistent with this observation made by others. In summary, our data suggested that dECM/PDA-coated PCL conduits were able to significantly enhance neuron-specific marker protein expression, and thus hold potential promise for future neurogenic applications. Therefore, these data also demonstrate that the use of 3D-printed dECM/PDA-coated nerve conduits could recruit moderate neuron-related growth factors for nerve regeneration. These results also suggest these 3D-printed dECM/PDA-coated nerve conduits are potentially suitable for large peripheral nerve defects in the future.

## 4. Conclusions 

In this study, we successfully fabricated a novel mussel-inspired bioactive dECM/PDA-coated PCL conduit for nerve regeneration. Our results showed that the conduits possessed strong mechanical properties when compared to the nerves of humans or animals. This property makes the conduit more tear-resistant for surgical procedures and handling. In addition, the presence of PDA improved the hydrophobicity of the conduit and increased the amount of dECM coating on the conduit. In vivo data demonstrated that such modifications demonstrated a higher level of laminins and collagens, which further enhanced cell adhesion, proliferation and differentiation as shown by the enhanced level of cell-adhered related proteins, cell morphology observations, enhanced ERK expressions and higher expression of cell-specific neuronal-markers Nestin, TUJ-1, MAP2 by RSC for nerve regeneration. Altogether, the current study proves that dECM/PDA-coated PCL conduits can function as a practical and clinically viable tool for promoting regenerative outcomes in larger peripheral nerve defects.
